# Bayesian adjustment for trend of colorectal cancer incidence in misclassified registering across Iranian provinces

**DOI:** 10.1371/journal.pone.0199273

**Published:** 2018-12-13

**Authors:** Sajad Shojaee, Nastaran Hajizadeh, Hadis Najafimehr, Luca Busani, Mohamad Amin Pourhoseingholi, Ahmad Reza Baghestani, Maryam Nasserinejad, Sara Ashtari, Mohammad Reza Zali

**Affiliations:** 1 Basic and Molecular Epidemiology of Gastrointestinal Disorders Research Center, Research Institute for Gastroenterology and Liver Diseases, Shahid Beheshti University of Medical Sciences, Tehran, Iran; 2 Physiotherapy Research Center, Department of Biostatistics, Faculty of Paramedical Sciences, Shahid Beheshti University of Medical Sciences, Tehran, Iran; 3 Gastroenterology and Liver Diseases Research Center, Research Institute for Gastroenterology and Liver Diseases, Shahid Beheshti University of Medical Sciences, Tehran, Iran; 4 Department of Infectious Diseases, Istituto Superiore di Sanità, Roma, Italy; Howard University, UNITED STATES

## Abstract

Misclassification error is a common problem of cancer registries in developing countries that leads to biased cancer rates. The purpose of this research is to use Bayesian method for correcting misclassification in registered cancer incidence of eighteen provinces in Iran. Incidence data of patients with colorectal cancer were extracted from Iranian annual of national cancer registration reports from 2005 to 2008. A province with proper medical facilities can always be compared to its neighbors. Almost 28% of the misclassification was estimated between the province of East Azarbaijan and West Azarbaijan, 56% between Fars and Hormozgan, 43% between Isfahan and Charmahal and Bakhtyari, 46% between Isfahan and Lorestan, 58% between Razavi Khorasan and North Khorasan, 50% between Razavi Khorasan and South Khorasan, 74% between Razavi Khorasan and Sistan and Balochestan, 43% between Mazandaran and Golestan, 37% between Tehran and Qazvin, 45% between Tehran and Markazi, 42% between Tehran and Qom, 47% between Tehran and Zanjan. Correcting the regional misclassification and obtaining the correct rates of cancer incidence in different regions is necessary for making cancer control and prevention programs and in healthcare resource allocation.

## Introduction

Colorectal cancer (CRC) is the third most common cancer among men (10.0% of the total) and the second in women (9.2% of the total) worldwide. Mortality is lower (694,000 deaths, 8.5% of the total) with more deaths (52%) in the less developed regions of the world, reflecting a poorer survival in these regions [1]. In Iran, CRC is the fourth most common type of cancer (the third most common cancer among females and the fifth among males), which accounts for 8.4% of total cancers in the country [2,3].

There is wide geographical variation in incidence across the world; the highest estimated rates is in Australia/New Zealand, and the lowest is in Western Africa. About 55% of the cases take place in more developed regions. Clearly, it is partly because of their advanced diagnostic and registration capabilities [1].

Inflammatory bowel disease, family history of CRC, obesity, dietary habits, smoking, physical inactivity [2,4], and diabetes [5] are well-known risk factors for CRC. Furthermore, environmental risk factors are found to play an important role in the incidence and development of CRC [4]. Therefore, people who live in the same or adjacent areas which are imposed on the same environmental risk factors are expected to have similar cancer incidence rates.

The population-based and accurate information on the occurrence of the cancer is extremely valuable as the foundation for identifying risk factors and making purposeful cancer prevention policies, because it is a leading cause of morbidity and mortality worldwide [6–8]. Cancer registries as the main sources of epidemiological data, collect information regarding the burden of cancers by recording the incidence, prevalence, survival and mortality of different cancers in a systematic manner [9–11]. Nowadays, their role has expanded into detecting the impact of interventions for cancer control, evaluation of screening programs, and specifying future needs for materials and manpower resources. However the existence of deficiencies in registering individual’s information including patient’s permanent residence, primary site of tumor, date of diagnosis, and date of death [8], makes the recorded data inaccurate to use in future studies.

In many developing countries like Iran, most cancer patients prefer to get diagnostic and medical treatment services in the capital or in their neighboring provinces, since health facilities are not distributed evenly throughout the country. [12]. Some patients never mention their permanent residence and are registered in those provinces. It causes misclassification error in cancer registry data. Misclassification error is the disagreement between the observed value and the true value in categorical data. The expected coverage of new cancer cases in different provinces can be mentioned as the evidence of existence of misclassification error in registering cancer incidence. The observed number of incidence is more than the expected number in some provinces, and on the other hand, it is less than expected in a neighboring province [13]. It occurs while it is expected that the rate of cancer incidence to be about the same in adjacent provinces; since people adopt very similar lifestyle and traditions and are exposed to same environmental conditions.

There are two approaches in correcting the misclassification error; the first approach is validating a small sample of data with rechecking medical records and extending the results to the target population [14]. The second approach is employing the Bayesian method. Bayesian method is a statistical approach that let us take our prior evidence into account [15] with determining prior information for some of the parameters [16–18].

The aim of this study is to investigate the trend of colorectal cancer provinces in Iran after estimating the misclassification rate in registering cancer incidence by using Bayesian method and re-estimating the incidence rate in each province.

## Material and methods

Registering of cancer reports is obtainable from different references such as pathologies, hospitals, death certificates and etc. National registration programming of cancer cases from Iranian annual of national cancer registration report is extracted during 2005 to 2008 with software which was created by health ministry, until cancer cases are collected, registered and centralized for the past couple of years and is used for data analyses. Hence all new diagnosed cancer cases in temporary information bank are sent from medical universities to ministry of health periodically. After process of duplicating and coding the recorded cancers based on 10th revision of international coding of disease, this information is registered in permanent information bank and all changes are sent to medical universities on specific duration, until permanent information bank of medical universities is equalized with permanent information bank of health ministry. So each medical university has an observed number of cancer cases and also has an expected coverage of cancer cases that are considered to be 100 per 100000 except 2008 that was 113 per 100000. By dividing the observed number to the expected number of cancer cases, the percent of expected coverage for each province is calculated [13].

Earlier this year, the national population-based cancer registry of the Islamic Republic of Iran was established, the International Agency for Research on Cancer (IARC) accepted Iran as a new Participating State and these registry data have been submitted to IARC to contribute to the next publications of GLOBOCAN and Cancer Incidence in Five Continents [19].

Since comparison of simple crude rate i.e. comparison of all cancer cases could make false images in total population regardless of age groups, age standardized rates (ASR) is calculated for all provinces of Iran using direct standardization method. The direct method for all provinces of Iran is based on, first selecting a criterion for the population and then calculating the desired outcome rate of this population using age specified rates at each of the two societies. At first, age groups were considered at level of 5 years. World standard population is the most common used standard population (*W*_*i*_). By dividing number of incident cases to person-years of observations, ASR is calculated per 100000 ($a_{i}=\frac{r_{i}}{n_{i}}$). Finally for 4 age groups(0–14 years, 15–49 years, 50–69 years and over than70 years old) and for both genders, ASR is calculated in order to compare statistics on cancer internationally(*ASR* = ∑_*i*_(*W*_*i*_×*a*_*i*_)) [20–22].

For entering the data to the Bayesian model two vectors *Y*_1_ and *Y*_2_ were used. Vector *Y*_1_ = [*Y*_11_,…,*Y*_*r*1_] for the province that has an expected coverage less than 100% with exact ASR and vector *Y*_2_ = [*Y*_12_,…,*Y*_*r*2_] for a neighboring province with a more than 100% expected coverage with ASR from the first group incorrectly labeled as being in the misclassified group. Subscript r is the number of covariate patterns for age and sex group combinations. A Poisson distribution was considered for count data *Y*_1_ and *Y*_2_ which first introduced by Stamey et al [20], then developed by Pourhoseingholi et al for mortality of cancer and also adopted by Hajizadeh et al for Iranian cancer incidence [22–24].

*Y*_1_ = *Poisson*(*P*_*i*_*μ*_*i*1_) and *Y*_2_ = *Poisson*(*P*_*i*_*μ*_*i*2_) in which *μ*_*i*1_ = *λ*_*i*1_(1−*θ*) and *μ*_*i*2_ = *λ*_*i*1_*θ*+*λ*_*i*2_ and the joint distribution of the count data *Y*_1_ and *Y*_2_ is proportional to:

$$\prod_{i=1}^{r}{\left[\lambda_{i1}(1-\theta )\right]}^{Y_{i1}}{\left[\lambda_{i1}\theta +\lambda_{i2}\right]}^{Y_{i2}}exp\{-P_{i}[\lambda_{i1}(1-\theta )]-P_{i}[\lambda_{i1}\theta +\lambda_{i2}]\}$$

An informative beta prior distribution was assumed for *θ* as the probability of a data from the first group incorrectly registered in the misclassified group; so *θ*~*Beta*(*a*,*b*). For selecting prior value for the parameters of beta distribution, the calculated expected coverage for the medical university which has a less than 100% expected coverage was used as *b* and *a* was calculated with subtracting *b* from 100. Thus *a*/(*a* + *b*) which is the expectation of beta distribution converges to the misclassified rate. Variable U with binomial distribution, i.e. *U*_*i*_|*Y*_1_,*Y*_2_,*θ*,*λ*_1_,*λ*_2_~*Binomial*(*Y*_*i*2_,*P*_*i*_) that $P_{i}=\frac{\lambda_{i1}\theta }{\lambda_{i1}\theta +\lambda_{i2}}$ was considered as the number of events from the first group that are incorrectly registered in the misclassified group. Now if *θ*, *Y*_1_, *Y*_2_ to be unknown; we have:

$$\pi (\theta |U_{i},Y_{1},Y_{2},\lambda_{1},\lambda_{2})=\pi (U_{i}|Y_{1},Y_{2},\theta ,\lambda_{1},\lambda_{2})\pi (Y_{1},Y_{2}|\theta ,\lambda_{1},\lambda_{2})\pi (\theta )$$

But since *Y*_1_, *Y*_2_ have known values of ASR on two neighboring provinces, then just theta is unknown and with employing a latent variable approach to correct the misclassification effect according to Paulino et al. [25,26], Liu et al. [27] and Stamey et al. [20] using a Gibbs sampling algorithm, the posterior appears in the following form:

$$\theta |U_{i},Y_{1},Y_{2},\lambda_{1},\lambda_{2}∼Beta\left(\sum_{i}U_{i}+a,\sum_{i}Y_{i1}+b\right)[22,24-28]$$

To determine the low facilitated provinces and the one to adjusted, a province with low-facility provinces (usually adjacent) with a coverage of less than 100 is considered, so that the province with a coverage of over 100 (almost in neighborhood) is adjusted. The low-facility was based on the local annual statistic report of unemployment, average income, etc.

After estimating the misclassification rate between each two neighboring provinces, the rates of colorectal cancer incidence for each province were re-estimated and the trend of colorectal cancer were carried out during 2005 to 2008. In order to perform the analyses the R software version 3.3.1 was used.

## Results

**Fig 1:**
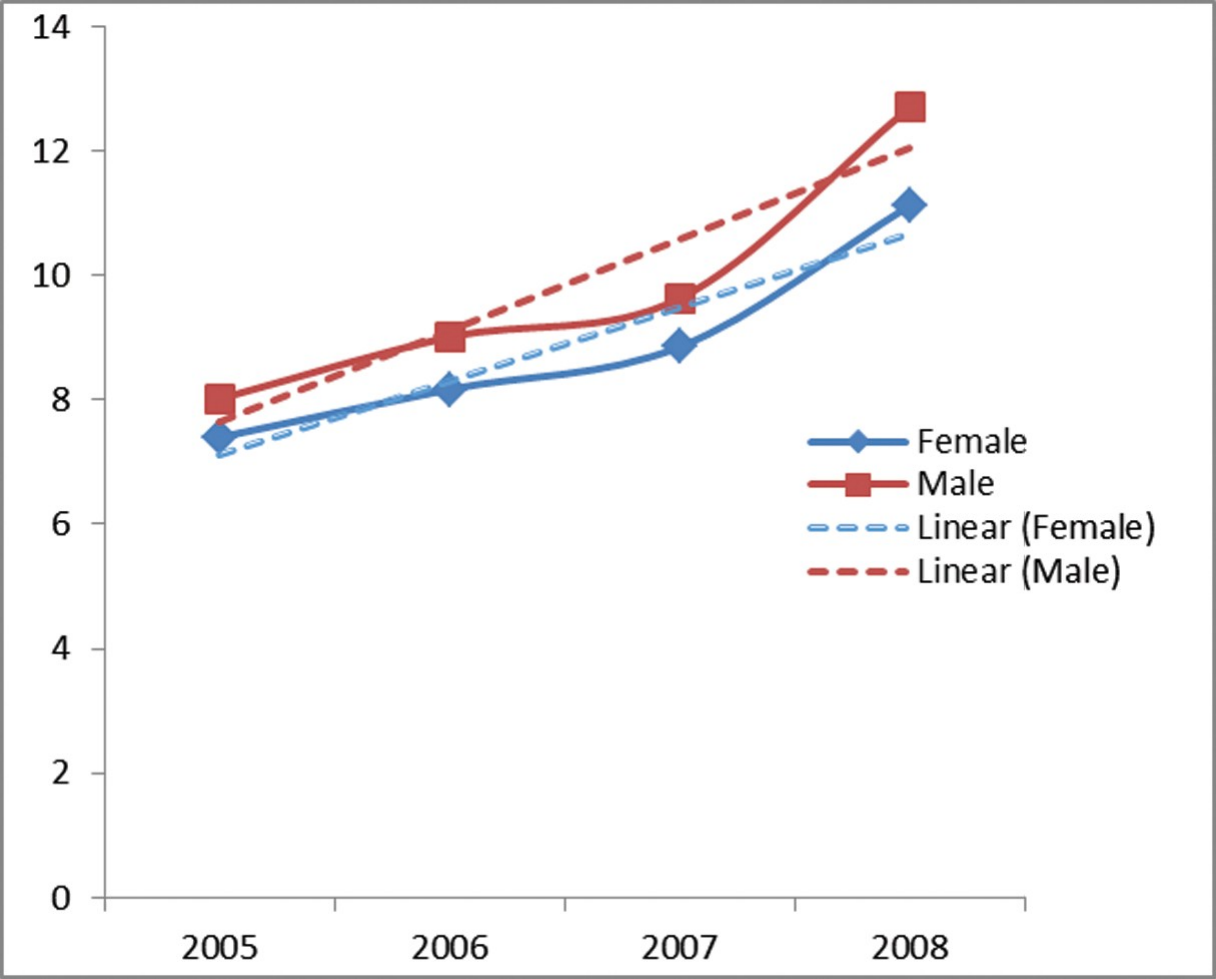
Age standardized rate of colorectal cancer incidence and its trend for male and female in Iran (2005–2008).

Registered cases of colorectal cancer have been included in the study for all provinces in Iran from 2005 to 2008. ASR of CRC incidence for men was 8.02 per 100,000 population (2255 cases) in 2005, whereas that year for women 7.4 per 100,000 (1801 cases). In over time, ASR of CRC incidence for men reached 12.7 per 100,000 population (3527 cases) in 2008 and for women to 11.12 per 100,000 (2658 cases) in the same year. The trend of CRC from 2005 to 2008 for both sexes is shown in Fig 1.

Among the 30 provinces, 18 provinces in which the number of cancer cases varied from their expected number were selected based on the percentage of expected cancer coverage, to correct the misclassification error in the registered data of neighboring provinces.

For example, the reported percentage of CRC expected coverage for Fars province as a province with suitable medical facilities and services was 120.8% in 2008. it means that Fars province have covered 20.8% of the new cases more than expected, while Hormozgan, which is adjacent to Fars, has a 19% expected coverage of cancer incidence, indicating clear misclassification in registering cancer cases. The expected coverage for all provinces in Iran between 2005 and 2008 is reported in Table 1. Also the estimated misclassification rate for all provinces in 2005–2008 is reported in Table 2.

**Table 1:** Expected coverage of cancer cases in provinces of Iran (2005–2008).

	2005	2006	2007	2008
East Azarbayejan	108.2	110.9	138.5	123.6
Isfahan	113.9	116.2	119.6	107.5
Razavi Khorasan	114.1	109.11	120.9	155.5
Tehran	157.11	162.25	145.7	155.6
Fars	84.2	119.4	143.3	120.8
Mazandaran	77	78	76.1	102.1
West Azarbayejan	81.9	75.3	82.5	69
Hormozgan	25.4	25.11	25.3	19
Chaharmahal	40.7	34.3	40.7	38
Lorestan	40.2	41.5	47.1	76
North Khorasan	30.8	40.4	44.8	34.8
South Khorasan	30.3	45.1	41.02	41.4
Sistan	27.2	19.1	18.85	19.5
Golestan	50.7	58.6	58.2	50.8
Qazvin	65.1	71.4	72.8	66.3
Markazi	43.3	53.06	57.4	69.6
Qom	38.6	62.7	60.9	53.9
zanjan	52.9	48.5	54.3	46.4

**Table 2:** Bayesian estimated from misclassification rate between provinces (2005–2008).

		Estimated misclassification rate			
		2005	2006	2007	2008
East Azarbayejan	West Azarbayejan	0.19	0.19	0.3	0.43
Fars	Hormozgan	0.45	0.61	0.58	0.58
Isfahan	Chaharmahal	0.44	0.43	0.38	0.47
Isfahan	Lorestan	0.52	0.51	0.51	0.32
Razavi Khorasan	North Khorasan	0.57	0.66	0.53	0.55
Razavi Khorasan	South Khorasan	0.6	0.3	0.56	0.55
Razavi Khorasan	Sistan		0.73	0.74	0.76
Mazandaran	Golestan	0.44	0.53	0.34	0.43
Tehran	Qazvin	0.33	0.36	0.36	0.43
Tehran	Markazi	0.49	0.44	0.41	0.45
Tehran	Qom	0.48	0.41	0.33	0.45
Tehran	zanjan	0.45	0.48	0.35	0.58

For example by using the Bayesian method, misclassification rate was estimated 58% between Fars and Hormozgan in 2008. So, after Bayesian correction, ASR and number of cancer incidence decrease for Fars province and increase for Hormozgan province. ASR and number of cancer incidence, before and after Bayesian correction from 2005 to 2008 are reported in Tables 3 and 4.

**Table 3:** Age standardized rate of colorectal cancer incidence before and after Bayesian correction in Iranian provinces 2005–2008.

	before Bayesian correction				after Bayesian correction			
	2005	2006	2007	2008	2005	2006	2007	2008
East Azarbayejan	3.52	3.26	10.68	14.15	2.51	1.95	8.60	11.15
Isfahan	8.19	9.26	9.05	9.54	4.74	5.00	5.78	4.14
Razavi Khorasan	6.10	9.44	11.04	12.96	3.53	5.32	7.04	9.65
Tehran	9.26	10.78	7.56	11.60	7.30	9.21	3.55	6.64
Fars	5.10	6.65	9.96	18.71	3.30	5.28	8.39	16.59
Mazandaran	9.03	10.08	10.36	12.76	6.30	6.20	13.32	9.21
West Azarbayejan	5.31	6.46	7.59	6.30	6.54	8.09	10.35	10.23
Hormozgan	3.46	3.00	4.19	4.99	9.59	10.29	13.80	20.23
Chaharmahal	6.00	7.83	10.15	7.55	12.49	17.65	19.63	16.88
Lorestan	3.96	4.52	5.45	9.52	9.08	10.07	11.35	13.53
North Khorasan	3.00	1.70	3.20	5.02	8.55	4.48	6.99	12.94
South Khorasan	2.48	2.89	2.88	4.40	7.39	4.81	6.81	10.24
Sistan	1.79	2.10	1.75	1.86	6.20	10.13	8.62	9.09
Golestan	5.44	7.69	9.12	7.73	10.16	14.65	14.45	14.27
Qazvin	7.92	6.66	5.80	9.23	11.93	10.02	8.67	15.22
Markazi	4.88	6.19	6.16	7.58	10.40	11.32	10.56	12.48
Qom	5.66	6.06	9.06	9.49	12.70	10.02	13.97	17.42
zanjan	5.43	5.38	9.21	5.23	10.05	10.70	15.15	11.77

**Table 4:** Number of colorectal cancer incidence and the percent of change before and after Bayesian correction in Iranian provinces 2005–2008.

	before Bayesian correction				after Bayesian correction			
	2005	2006	2007	2008	2005	2006	2007	2008
East Azarbayejan	95	85	293	379	68	51	236	299
Isfahan	268	312	279	302	155	168	178	131
Razavi Khorasan	307	396	377	432	178	223	241	322
Tehran	819	1034	315	481	646	883	148	276
Fars	166	212	951	1870	108	132	801	1658
Mazandaran	192	214	217	274	134	132	279	198
West Azarbayejan	118	135	157	129	145	169	214	209
Hormozgan	33	33	44	56	91	113	145	227
Chaharmahal	41	46	65	50	85	104	126	112
Lorestan	53	70	70	115	122	156	146	163
North Khorasan	19	10	18	31	54	26	39	80
South Khorasan	9	11	12	18	27	18	28	42
Sistan	31	39	33	34	107	188	163	167
Golestan	67	91	106	90	125	173	168	166
Qazvin	59	57	48	75	89	86	72	124
Markazi	49	65	63	76	104	119	108	125
Qom	44	47	72	74	99	78	111	136
zanjan	39	38	66	42	72	76	109	95

## Discussion

It is obvious that the neighboring provinces due to the same eating habits, lifestyle and climate, have the same health outcomes [13]. But sometimes when analyzing registered data, it is observed that the neighboring provinces not only have different outcomes but are also inconsistent. This situation implies that there is misclassification in registered data. This problem is a notable matter in medicine which may results in deflecting of health programing and health resources allocation [28]. Such deflection could make irrecoverable damage on national scale. The aim of the present study was to help reducing misclassification error in registered colorectal cancer data in Iran. Firstly, the means in accessing health resources in welfare provinces and secondly the lack of health facilities in their neighboring provinces are elements in creating misclassification error. Fortunately, some studies have been conducted in Iran in order to eliminate the misclassification errors for mortality and morbidity registered cancer data in the case of Liver [29], Gastric [22], and colorectal cancers [23]. Applying the results of the studies above may be more reliable, since they had re-estimated and produced valid data. According to the result of our research, there was a non-ignorable estimated misclassification rate among adjacent provinces. The highest estimated misclassification parameter, belongs to North Khorasan, Hormozgan, and Sistan which are in the east and south of Iran. So the real rates of CRC in those provinces are higher than the rates that are reported by cancer registry system.

On the contrary, in studies that use cancer registry data and ignore the existence of misclassification error, it is reported that the highest incidence rates of CRC in Iran were found in the central, northern, and western provinces; and the southwest provinces of Iran had the lowest incidence rates of CRC in the country! [2]. Therefore, ignoring the misclassification error in registered data, leads to a wrong image of distribution of CRC incidence across the country. Expected cancer coverage revealed that from 30 provinces, 18 provinces need misclassification correction. These provinces are those which are different in economic situation and there are some points in them which are welfare and probably patients for better health care, refers to those welfare places, so they have more referring people than their capacity. On the other hand, some provinces due to their fewer facilities, have fewer referring patients. Table 2 is indicating how the data of some provinces are registered in their adjacent locations. For example, some neighboring Tehrani patients like Qom’s patients, were referred to Tehran.

**Fig 2:**
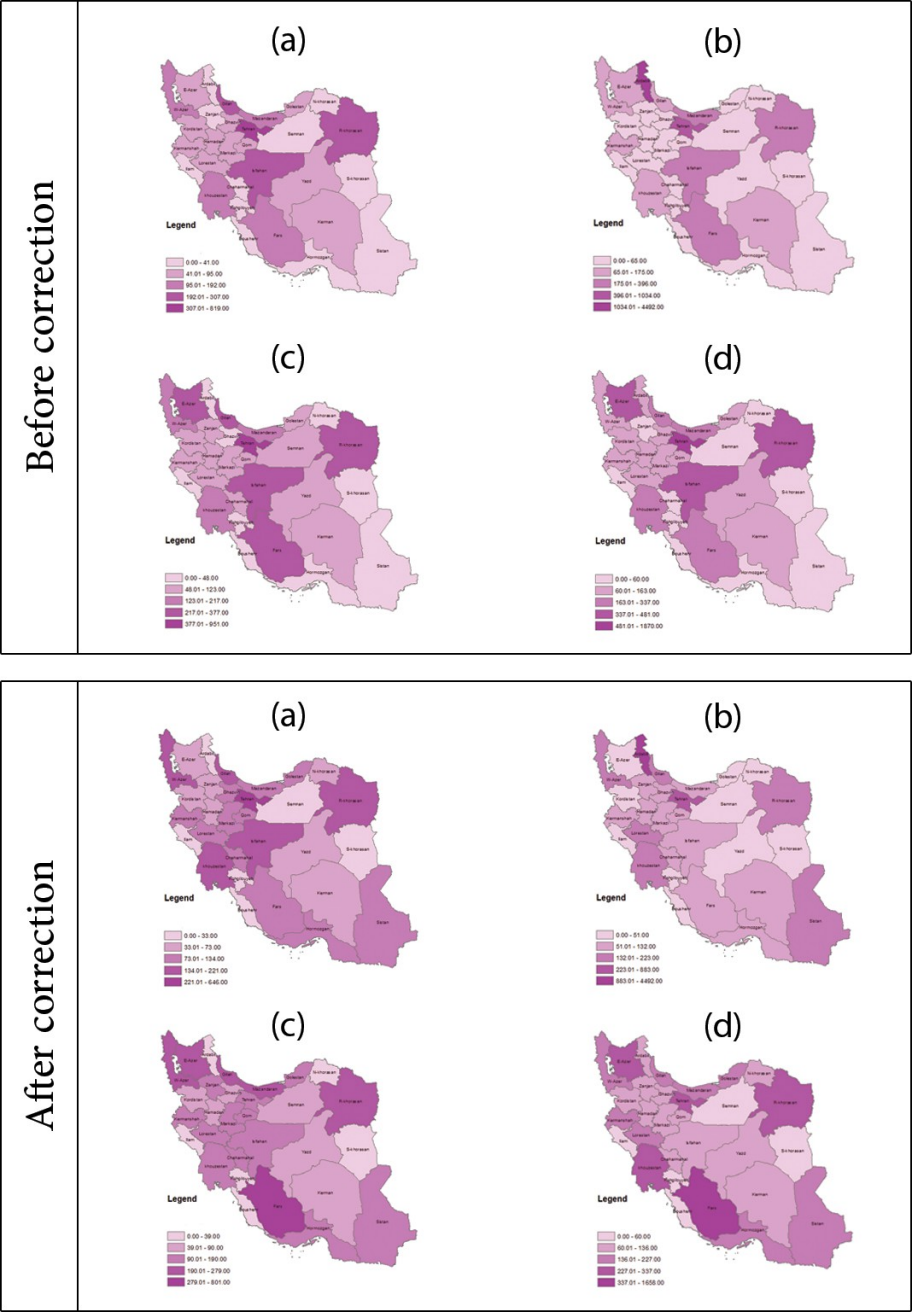
Distribution of colorectal cancer incidence in Iran before and after misclassification error correction since 2005 to 2009. (a) 2005, (b) 2006, (c) 2007, (d) 2008.

Identifying the exact distribution of a disease in different areas is a suitable way for finding the geographic pattern of the disease and causations, assessing the influential factors on disease incidence [30,31], and quantifying the potentials for disease control and prevention [32,33]. However, spatial analysis is usually deployed for this purpose which is based on registered data while existence of misclassification is often ignored. In spatial analysis, the morbidity or mortality rates for each province are combined with local information for the same province and the result may lead to an integrated geographical map. This type of maps is helpful for comparing among different provinces in aspect of disease incidence rate or probable risk factors [34]. In order to achieve this goal, we have prepared geographical map to evaluate incidence distribution of colorectal cancer registered data for before and after misclassification correction in Fig 2. Fig 2 showed that after correction the southern provinces have high incidence rate, while in the previous studies that ignored misclassification, southern provinces had low incidence rate [35].

The maps of present study also revealed that considerable changes happened in some provinces respect to before correction status. Thus, there are major differences in the incidence of CRC, while it is expected that the incidence of cancer to be the same in adjacent provinces. This can be justified by existence of misclassification error in registering permanent address of patients who are diagnosed in neighboring facilitate provinces. It leads to overestimation of CRC rate in some provinces and underestimation of its rate in some neighboring provinces.

For future researches, to recognize high risk spatial clusters, using our colorectal cancer valid data, is suggested. Also we could comparison and validate the information from this study in misclassification, with random sample of provinces in the future studies.

In conclusion, proper planning for cancer control and prevention, and allocating healthcare facilities to different areas, requires an increase in the quality and accuracy of registering system in different provinces and the correction of the existing deficiencies especially misclassification error in registering patient’s permanent residence. The hardware and software resources need to be enhanced, more educated staff need to be trained in different sectors of cancer registry program, and the opinions of expert researchers in medicine, biostatistics and epidemiology need to be implemented [36]. In the absence of valid data, Bayesian method can be adopted as a fast and cost effective method to correct the regional misclassification error.

## Supporting information

S1 File The original data of colorectal cancer incidence for both sexes, and age group, and calculated ASR, for all Iranian provinces which included in this study, 2005–2008. (RAR)
